# 1-Nitro-2,3-di-2-pyridyl-2,3-dihydro­indolizine

**DOI:** 10.1107/S1600536809011842

**Published:** 2009-04-02

**Authors:** Martin Schulz, Tobias Kloubert, Helmar Görls, Matthias Westerhausen

**Affiliations:** a Institute of Inorganic and Analytical Chemistry, Friedrich-Schiller-Universität Jena, August-Bebel-Strasse 2, D-07743 Jena, Germany

## Abstract

The title compound, C_18_H_14_N_4_O_2_, was found as a by-product in the nitro­aldol reaction between 2-(nitro­meth­yl)pyridine and *N*-(pyridin-2-ylmethyl­idene)methane­amine. Its two stereogenic centers give rise to four stereoisomers of which only the *anti* isomers are found in this crystal structure.

## Related literature

For the synthesis of 2-(nitro­meth­yl)pyridine, see: Feuer & Lawrence (1972 ▶). For nitro­aldol reactions, see: Cwik *et al.* (2005 ▶). For β-nitro­amines, see Lucet *et al.* (1998 ▶). For comparison of bond lengths, see: Allen *et al.* (1987 ▶).
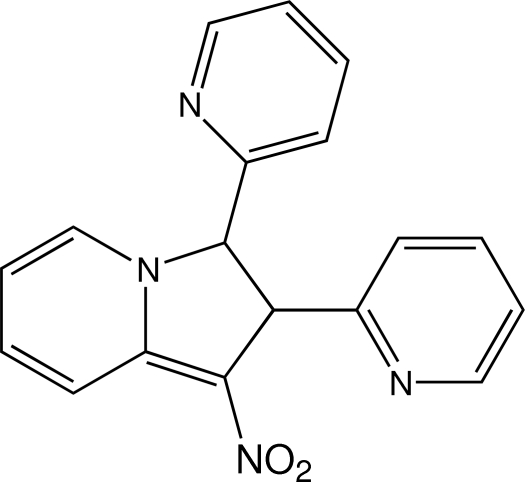


## Experimental

### 

#### Crystal data

C_18_H_14_N_4_O_2_
*M* *_r_* = 318.33
Monoclinic, 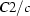

*a* = 28.0688 (19) Å
*b* = 7.9672 (6) Å
*c* = 21.1859 (15) Å
β = 131.408 (4)°
*V* = 3553.4 (4) Å^3^
*Z* = 8
Mo *K*α radiation
μ = 0.08 mm^−1^
*T* = 183 K
0.06 × 0.06 × 0.05 mm

#### Data collection

Nonius KappaCCD diffractometer
Absorption correction: none
10925 measured reflections
4021 independent reflections
2564 reflections with *I* > 2σ(*I*)
*R*_int_ = 0.057

#### Refinement

*R*[*F* ^2^ > 2σ(*F* ^2^)] = 0.065
*wR*(*F* ^2^) = 0.216
*S* = 0.74
4021 reflections
217 parameters
H-atom parameters constrained
Δρ_max_ = 0.28 e Å^−3^
Δρ_min_ = −0.26 e Å^−3^

### 

Data collection: *COLLECT* (Nonius, 1998 ▶); cell refinement: *DENZO* (Otwinowski & Minor, 1997 ▶); data reduction: *DENZO* and the SQUEEZE option (Sluis & Spek, 1990 ▶) in *PLATON* (Spek, 2009 ▶); program(s) used to solve structure: *SHELXS97* (Sheldrick, 2008 ▶); program(s) used to refine structure: *SHELXL97* (Sheldrick, 2008 ▶); molecular graphics: *SHELXTL/PC* (Sheldrick, 2008 ▶); software used to prepare material for publication: *SHELXL97*.

## Supplementary Material

Crystal structure: contains datablocks I, global. DOI: 10.1107/S1600536809011842/bt2909sup1.cif

Structure factors: contains datablocks I. DOI: 10.1107/S1600536809011842/bt2909Isup2.hkl

Additional supplementary materials:  crystallographic information; 3D view; checkCIF report

## Figures and Tables

**Fig. 1.**
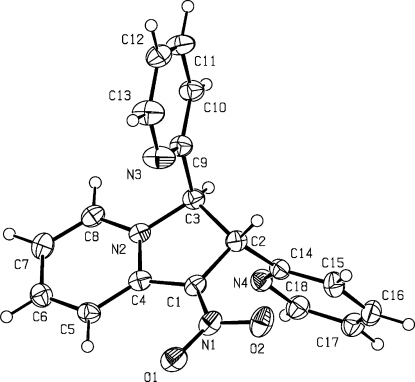
Molecular structure an numbering scheme of 3. The ellipsoids represent a probability of 40%, H atoms are shown with arbitrary radii.

**Fig. 2.**
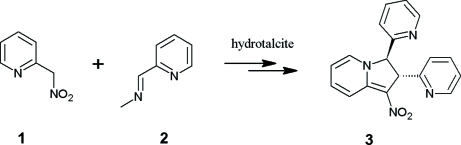
The formation of the title compound.

## supplementary crystallographic information

### Comment

β-nitroamines are promising precursors for vicinal diamines, which themselves are a versatile class of compounds (Lucet *et al.*, 1998). The nitroaldol reaction between 2-(nitromethyl)pyridine 1 (Feuer & Lawrence, 1972) and *N*-(pyridin-2-ylmethylidene)methaneamine 2 yielded the title compound 3 as a byproduct together with methylamine. Although four stereo isomers are possible, only the anti-isomers are found in the crystal structure. The 2-pyridyl rings of neighbouring molecules are arrangend coplanarily with an approximate intermolecular distance of 3.70 Å. Not surprisingly the five-membered dihydroindolizine ring is strained and conjugation of the six-membered ring with the nitro group is observed regarding the bond lengths. They are found to lie between those for single and double bonds (Allen *et al.*, 1987).

### Experimental

A nitroaldol reaction (Henry reaction) was carried out with 2-(nitromethyl)pyridine 1 (0.20 g; 1.4 mmol) and *N*-(pyridin-2-ylmethylidene)methaneamine 2 (0.15 g; 1.2 mmol) in 3.5 ml anhydrous THF with hydrotalcite Syntal 696 (0.13 g) as catalyst (Cwik *et al.*, 2005). The mixture was stirred for eight hours at 60 °C and then cooled to r.t.. Then the solvent was removed *in vacuo* yielding a sticky brown residue. Thereafter 3 ml of diethylether were added and the yellow etheral solution was separated from the insoluble brownish residue, which was then dissolved in THF. From the latter solution yellow crystals of the title compound were obtained at r.t. (0.017 g). The compound is stable at room temperature and under atmospheric conditions. NMR measurements were carried out on a Bruker AC 200 and Bruker AC 400 spectrometer and referenced to the solvent resonances. Signals were assigned by DEPT 135, HSQC, HMBC experiments.

^1^H-NMR (CD_2_Cl_2_): δ = 8.59 (d, 1H, J = 4.0 Hz, H13), 8.55 (d, 1H, J = 4.0 Hz, H18), 8.30 (d, 1H, J = 8.8 Hz, H5), 7.80–7.77 (m, 1H, H11), 7.76–7.73 (m, 1H, H6), 7.69–7.65 (m, 1H, H16), 7.58 (d, 1H, J = 6.4 Hz, H8), 7.43 (d, 1H, J = 7.6 Hz, H12), 7.35–7.33 (m, 1H, H15), 7.33–7.31(m, 1H, H17), 7.23–7.20 (m, 1H, H19), 6.74–6.71 (m, 1H, H7), 6.08 (d, 1H, J = 4.0 Hz, H3), 4.89 (d, 1H, J = 4.0 Hz, H2);

^13^C-NMR (CD_2_Cl_2_): δ = 159.7 (q,C14), 157.5 (q,C4,C9), 150.7 (t,C13), 150.4 (q,C1), 150.1(t,C18), 142.2 (t,C6), 138.0 (t,C11), 137.3 (t,C8), 136.7 (t,C16), 124.5 (t,C15), 124.1 (t,C12), 122.8 (t,C10), 121.8 (t,C17), 119.6 (t,C5), 114.8 (t,C7), 75.3 (t,C3), 54.1 (t,C2).

### Refinement

All hydrogen atoms were calculated at idealized positions and were refined with 1.2 times the isotropic displacement parameter of the corresponding carbon atoms. At the final stage of refinement, clear evidence of the presence of solvent voids of 201.00 Å ^3^ was obtained. Several trials to find a reasonable model for this were unfruitful. Thus, a correction for diffuse effects due to the inclusion of disordered solvent molecules in the crystal structure was made using the the SQUEEZE option (van der Sluis & Spek, 1990) in the program PLATON (Spek, 2009). Further details are given in the cif.

### Crystal data

**Table:**

C_18_H_14_N_4_O_2_	*F*(000) = 1364
*M**_r_* = 318.33	*D*_x_ = 1.219 Mg m^−^^3^
Monoclinic, *C*2/*c*	Mo *K*α radiation, λ = 0.71073 Å
Hall symbol: -C 2yc	Cell parameters from 10925 reflections
*a* = 28.0688 (19) Å	θ = 2.6–27.5°
*b* = 7.9672 (6) Å	µ = 0.08 mm^−^^1^
*c* = 21.1859 (15) Å	*T* = 183 K
β = 131.408 (4)°	Prism, light yellow
*V* = 3553.4 (4) Å^3^	0.06 × 0.06 × 0.05 mm
*Z* = 8	

### Data collection

**Table:**

Nonius KappaCCD diffractometer	2564 reflections with *I* > 2σ(*I*)
Radiation source: fine-focus sealed tube	*R*_int_ = 0.057
graphite	θ_max_ = 27.5°, θ_min_ = 2.6°
φ and ω scans	*h* = −36→36
10925 measured reflections	*k* = −10→8
4021 independent reflections	*l* = −22→27

### Refinement

**Table:**

Refinement on *F*^2^	Primary atom site location: structure-invariant direct methods
Least-squares matrix: full	Secondary atom site location: difference Fourier map
*R*[*F*^2^ > 2σ(*F*^2^)] = 0.065	Hydrogen site location: inferred from neighbouring sites
*wR*(*F*^2^) = 0.216	H-atom parameters constrained
*S* = 0.74	*w* = 1/[σ^2^(*F*_o_^2^) + (0.1821*P*)^2^ + 4.6408*P*] where *P* = (*F*_o_^2^ + 2*F*_c_^2^)/3
4021 reflections	(Δ/σ)_max_ < 0.001
217 parameters	Δρ_max_ = 0.28 e Å^−^^3^
0 restraints	Δρ_min_ = −0.26 e Å^−^^3^

### Special details

**Table:**

Geometry. All e.s.d.'s (except the e.s.d. in the dihedral angle between two l.s. planes) are estimated using the full covariance matrix. The cell e.s.d.'s are taken into account individually in the estimation of e.s.d.'s in distances, angles and torsion angles; correlations between e.s.d.'s in cell parameters are only used when they are defined by crystal symmetry. An approximate (isotropic) treatment of cell e.s.d.'s is used for estimating e.s.d.'s involving l.s. planes.
Refinement. Refinement of *F*^2^ against ALL reflections. The weighted *R*-factor *wR* and goodness of fit *S* are based on *F*^2^, conventional *R*-factors *R* are based on *F*, with *F* set to zero for negative *F*^2^. The threshold expression of *F*^2^ > σ(*F*^2^) is used only for calculating *R*-factors(gt) *etc*. and is not relevant to the choice of reflections for refinement. *R*-factors based on *F*^2^ are statistically about twice as large as those based on *F*, and *R*- factors based on ALL data will be even larger.

### Fractional atomic coordinates and isotropic or equivalent isotropic displacement parameters (Å^2^)

**Table:**

	*x*	*y*	*z*	*U*_iso_*/*U*_eq_	
O1	0.17598 (9)	1.2960 (2)	0.14775 (13)	0.0452 (5)	
O2	0.16787 (9)	1.2549 (2)	0.03788 (12)	0.0485 (5)	
N1	0.17260 (9)	1.1975 (2)	0.09773 (14)	0.0371 (5)	
N2	0.17010 (8)	0.7804 (2)	0.15264 (11)	0.0298 (4)	
N3	0.04544 (10)	0.8231 (3)	−0.00295 (16)	0.0518 (6)	
N4	0.27897 (9)	0.8055 (3)	0.13515 (12)	0.0357 (5)	
C1	0.17419 (10)	1.0305 (3)	0.10757 (14)	0.0325 (5)	
C2	0.17024 (10)	0.9055 (3)	0.05051 (14)	0.0323 (5)	
H2A	0.1348	0.9368	−0.0097	0.039*	
C3	0.15344 (10)	0.7422 (3)	0.07139 (14)	0.0320 (5)	
H3A	0.1790	0.6456	0.0776	0.038*	
C4	0.17739 (10)	0.9488 (3)	0.16928 (13)	0.0309 (5)	
C5	0.18579 (10)	1.0032 (3)	0.23890 (14)	0.0338 (5)	
H5A	0.1914	1.1190	0.2528	0.041*	
C6	0.18587 (11)	0.8876 (3)	0.28665 (15)	0.0395 (6)	
H6A	0.1912	0.9242	0.3337	0.047*	
C7	0.17820 (12)	0.7160 (3)	0.26747 (16)	0.0408 (6)	
H7A	0.1781	0.6365	0.3008	0.049*	
C8	0.17096 (11)	0.6654 (3)	0.20010 (15)	0.0349 (5)	
H8A	0.1665	0.5496	0.1866	0.042*	
C9	0.08294 (11)	0.7031 (3)	0.00711 (14)	0.0344 (5)	
C10	0.06039 (11)	0.5554 (3)	−0.03744 (15)	0.0385 (6)	
H10A	0.0886	0.4714	−0.0278	0.046*	
C11	−0.00528 (12)	0.5328 (3)	−0.09736 (17)	0.0473 (7)	
H11A	−0.0227	0.4328	−0.1299	0.057*	
C12	−0.04402 (12)	0.6548 (4)	−0.10870 (16)	0.0451 (6)	
H12A	−0.0889	0.6421	−0.1494	0.054*	
C13	−0.01697 (13)	0.7973 (4)	−0.0600 (2)	0.0525 (7)	
H13A	−0.0443	0.8817	−0.0675	0.063*	
C14	0.23253 (10)	0.8989 (3)	0.06895 (13)	0.0317 (5)	
C15	0.24114 (12)	0.9906 (3)	0.02183 (15)	0.0394 (6)	
H15A	0.2071	1.0545	−0.0250	0.047*	
C16	0.29952 (13)	0.9886 (3)	0.04343 (17)	0.0444 (6)	
H16A	0.3066	1.0520	0.0123	0.053*	
C17	0.34797 (12)	0.8915 (3)	0.11200 (17)	0.0437 (6)	
H17A	0.3887	0.8865	0.1286	0.052*	
C18	0.33500 (12)	0.8037 (3)	0.15471 (16)	0.0401 (6)	
H18A	0.3680	0.7373	0.2013	0.048*	

### Atomic displacement parameters (Å^2^)

**Table:**

	*U*^11^	*U*^22^	*U*^33^	*U*^12^	*U*^13^	*U*^23^
O1	0.0509 (11)	0.0294 (9)	0.0678 (12)	0.0003 (7)	0.0446 (10)	−0.0052 (8)
O2	0.0574 (12)	0.0393 (10)	0.0642 (12)	0.0097 (8)	0.0467 (11)	0.0178 (9)
N1	0.0373 (11)	0.0281 (10)	0.0527 (12)	0.0046 (8)	0.0327 (10)	0.0056 (9)
N2	0.0270 (9)	0.0276 (10)	0.0322 (9)	−0.0005 (7)	0.0185 (8)	0.0000 (7)
N3	0.0335 (11)	0.0431 (13)	0.0634 (14)	0.0014 (9)	0.0254 (11)	−0.0136 (11)
N4	0.0336 (10)	0.0381 (11)	0.0356 (10)	−0.0006 (8)	0.0229 (9)	0.0012 (8)
C1	0.0313 (11)	0.0288 (12)	0.0382 (12)	0.0004 (9)	0.0233 (10)	0.0015 (9)
C2	0.0326 (11)	0.0305 (12)	0.0303 (10)	0.0002 (9)	0.0193 (10)	0.0020 (9)
C3	0.0313 (11)	0.0285 (11)	0.0366 (11)	0.0008 (9)	0.0226 (10)	−0.0028 (9)
C4	0.0253 (10)	0.0260 (11)	0.0363 (11)	0.0020 (8)	0.0182 (9)	0.0006 (9)
C5	0.0335 (11)	0.0298 (12)	0.0377 (12)	0.0009 (9)	0.0234 (10)	−0.0031 (9)
C6	0.0384 (13)	0.0440 (15)	0.0386 (12)	0.0032 (10)	0.0266 (11)	0.0006 (10)
C7	0.0402 (13)	0.0415 (14)	0.0443 (13)	0.0045 (10)	0.0295 (12)	0.0092 (11)
C8	0.0333 (12)	0.0282 (12)	0.0420 (12)	0.0001 (9)	0.0244 (11)	0.0026 (10)
C9	0.0324 (12)	0.0328 (12)	0.0371 (12)	0.0001 (9)	0.0226 (10)	−0.0013 (9)
C10	0.0377 (12)	0.0325 (13)	0.0449 (13)	−0.0023 (10)	0.0272 (11)	−0.0042 (10)
C11	0.0372 (13)	0.0426 (15)	0.0526 (15)	−0.0113 (11)	0.0256 (12)	−0.0134 (12)
C12	0.0303 (12)	0.0531 (16)	0.0452 (14)	−0.0063 (11)	0.0221 (11)	−0.0039 (12)
C13	0.0345 (13)	0.0471 (16)	0.0651 (17)	0.0043 (11)	0.0284 (13)	−0.0086 (13)
C14	0.0352 (12)	0.0307 (12)	0.0305 (10)	−0.0021 (9)	0.0223 (10)	−0.0032 (9)
C15	0.0469 (14)	0.0398 (14)	0.0369 (12)	0.0043 (11)	0.0299 (12)	0.0055 (10)
C16	0.0566 (16)	0.0427 (15)	0.0532 (15)	−0.0055 (12)	0.0445 (14)	−0.0008 (12)
C17	0.0408 (13)	0.0468 (15)	0.0539 (15)	−0.0062 (11)	0.0357 (13)	−0.0068 (12)
C18	0.0357 (12)	0.0430 (14)	0.0410 (13)	0.0024 (10)	0.0252 (11)	0.0015 (10)

### Geometric parameters (Å, °)

**Table:**

O1—N1	1.271 (3)	C6—H6A	0.9500
O2—N1	1.269 (3)	C7—C8	1.363 (4)
N1—C1	1.342 (3)	C7—H7A	0.9500
N2—C8	1.349 (3)	C8—H8A	0.9500
N2—C4	1.367 (3)	C9—C10	1.373 (3)
N2—C3	1.487 (3)	C10—C11	1.395 (3)
N3—C13	1.331 (3)	C10—H10A	0.9500
N3—C9	1.331 (3)	C11—C12	1.356 (4)
N4—C18	1.335 (3)	C11—H11A	0.9500
N4—C14	1.342 (3)	C12—C13	1.377 (4)
C1—C4	1.410 (3)	C12—H12A	0.9500
C1—C2	1.513 (3)	C13—H13A	0.9500
C2—C14	1.519 (3)	C14—C15	1.381 (3)
C2—C3	1.545 (3)	C15—C16	1.380 (4)
C2—H2A	1.0000	C15—H15A	0.9500
C3—C9	1.517 (3)	C16—C17	1.394 (4)
C3—H3A	1.0000	C16—H16A	0.9500
C4—C5	1.400 (3)	C17—C18	1.370 (4)
C5—C6	1.367 (3)	C17—H17A	0.9500
C5—H5A	0.9500	C18—H18A	0.9500
C6—C7	1.402 (4)		
			
O1—N1—O2	120.7 (2)	C6—C7—H7A	120.7
O1—N1—C1	120.4 (2)	C7—C8—N2	119.8 (2)
O2—N1—C1	118.9 (2)	C7—C8—H8A	120.1
C8—N2—C4	123.25 (19)	N2—C8—H8A	120.1
C8—N2—C3	124.12 (19)	N3—C9—C10	123.3 (2)
C4—N2—C3	112.24 (18)	N3—C9—C3	114.8 (2)
C13—N3—C9	117.5 (2)	C10—C9—C3	121.8 (2)
C18—N4—C14	117.4 (2)	C9—C10—C11	117.9 (2)
N1—C1—C4	125.2 (2)	C9—C10—H10A	121.1
N1—C1—C2	123.4 (2)	C11—C10—H10A	121.1
C4—C1—C2	111.29 (19)	C12—C11—C10	119.4 (2)
C1—C2—C14	110.71 (18)	C12—C11—H11A	120.3
C1—C2—C3	101.58 (17)	C10—C11—H11A	120.3
C14—C2—C3	114.49 (18)	C11—C12—C13	118.6 (2)
C1—C2—H2A	109.9	C11—C12—H12A	120.7
C14—C2—H2A	109.9	C13—C12—H12A	120.7
C3—C2—H2A	109.9	N3—C13—C12	123.4 (2)
N2—C3—C9	107.82 (17)	N3—C13—H13A	118.3
N2—C3—C2	103.72 (17)	C12—C13—H13A	118.3
C9—C3—C2	112.33 (18)	N4—C14—C15	122.4 (2)
N2—C3—H3A	110.9	N4—C14—C2	116.42 (19)
C9—C3—H3A	110.9	C15—C14—C2	121.2 (2)
C2—C3—H3A	110.9	C16—C15—C14	119.4 (2)
N2—C4—C5	117.9 (2)	C16—C15—H15A	120.3
N2—C4—C1	107.89 (19)	C14—C15—H15A	120.3
C5—C4—C1	134.2 (2)	C15—C16—C17	118.6 (2)
C6—C5—C4	119.2 (2)	C15—C16—H16A	120.7
C6—C5—H5A	120.4	C17—C16—H16A	120.7
C4—C5—H5A	120.4	C18—C17—C16	118.0 (2)
C5—C6—C7	121.2 (2)	C18—C17—H17A	121.0
C5—C6—H6A	119.4	C16—C17—H17A	121.0
C7—C6—H6A	119.4	N4—C18—C17	124.2 (2)
C8—C7—C6	118.7 (2)	N4—C18—H18A	117.9
C8—C7—H7A	120.7	C17—C18—H18A	117.9
			
O1—N1—C1—C4	2.2 (3)	C6—C7—C8—N2	−1.2 (3)
O2—N1—C1—C4	−178.0 (2)	C4—N2—C8—C7	1.3 (3)
O1—N1—C1—C2	179.84 (19)	C3—N2—C8—C7	−170.9 (2)
O2—N1—C1—C2	−0.4 (3)	C13—N3—C9—C10	−0.8 (4)
N1—C1—C2—C14	74.7 (3)	C13—N3—C9—C3	178.4 (2)
C4—C1—C2—C14	−107.4 (2)	N2—C3—C9—N3	56.2 (3)
N1—C1—C2—C3	−163.3 (2)	C2—C3—C9—N3	−57.5 (3)
C4—C1—C2—C3	14.6 (2)	N2—C3—C9—C10	−124.6 (2)
C8—N2—C3—C9	69.8 (3)	C2—C3—C9—C10	121.8 (2)
C4—N2—C3—C9	−103.2 (2)	N3—C9—C10—C11	1.4 (4)
C8—N2—C3—C2	−170.94 (19)	C3—C9—C10—C11	−177.8 (2)
C4—N2—C3—C2	16.1 (2)	C9—C10—C11—C12	−0.7 (4)
C1—C2—C3—N2	−17.3 (2)	C10—C11—C12—C13	−0.4 (4)
C14—C2—C3—N2	102.0 (2)	C9—N3—C13—C12	−0.5 (5)
C1—C2—C3—C9	98.8 (2)	C11—C12—C13—N3	1.0 (5)
C14—C2—C3—C9	−141.83 (19)	C18—N4—C14—C15	0.1 (3)
C8—N2—C4—C5	−0.3 (3)	C18—N4—C14—C2	−177.2 (2)
C3—N2—C4—C5	172.68 (18)	C1—C2—C14—N4	80.8 (2)
C8—N2—C4—C1	179.81 (18)	C3—C2—C14—N4	−33.3 (3)
C3—N2—C4—C1	−7.2 (2)	C1—C2—C14—C15	−96.5 (2)
N1—C1—C4—N2	172.5 (2)	C3—C2—C14—C15	149.4 (2)
C2—C1—C4—N2	−5.3 (2)	N4—C14—C15—C16	−0.7 (4)
N1—C1—C4—C5	−7.3 (4)	C2—C14—C15—C16	176.4 (2)
C2—C1—C4—C5	174.8 (2)	C14—C15—C16—C17	0.8 (4)
N2—C4—C5—C6	−0.5 (3)	C15—C16—C17—C18	−0.4 (4)
C1—C4—C5—C6	179.3 (2)	C14—N4—C18—C17	0.4 (4)
C4—C5—C6—C7	0.5 (3)	C16—C17—C18—N4	−0.3 (4)
C5—C6—C7—C8	0.4 (4)		
